# Hypoxia promotes colon cancer dissemination through up-regulation of cell migration-inducing protein (CEMIP)

**DOI:** 10.18632/oncotarget.3978

**Published:** 2015-05-13

**Authors:** Nikki A. Evensen, Yiyi Li, Cem Kuscu, Jingxuan Liu, Jillian Cathcart, Anna Banach, Qian Zhang, Ellen Li, Sonia Joshi, Jie Yang, Paula I Denoya, Silvia Pastorekova, Stanley Zucker, Kenneth R. Shroyer, Jian Cao

**Affiliations:** ^1^ Department of Medicine/Division of Cancer Prevention, Stony Brook University, Stony Brook, NY 11794, USA; ^2^ Department of Pathology, Stony Brook University, Stony Brook, NY 11794, USA; ^3^ Department of Medicine/Division of Gastroenterology, Stony Brook University, Stony Brook, NY 11794, USA; ^4^ Department of Preventative Medicine, Stony Brook University, Stony Brook, NY 11794, USA; ^5^ Department of Surgery, Stony Brook University, Stony Brook, NY 11794, USA; ^6^ Department of Molecular Medicine, Institute of Virology, Slovak Academy of Sciences, Bratislava 84505, Slovak Republic; ^7^ Veterans Affairs Medical Center, Northport, NY 11768, USA; ^8^ Department of Radiation Oncology, Nanfang Hospital, Southern Medical University, Guangzhou 510515, China; ^9^ Department of Pediatrics, NYU Medical School, New York, NY 10016, USA; ^10^ Department of Biochemistry and Molecular Genetics, University of Virginia, Charlottesville, VA 22908, USA

**Keywords:** KIAA1199/CEMIP, migration, invasion, HIF-2α

## Abstract

Hypoxic stress drives cancer progression by causing a transcriptional reprogramming. Recently, KIAA1199 was discovered to be a *ce*ll-*m*igration *i*nducing *p*rotein (renamed CEMIP) that is upregulated in human cancers. However, the mechanism of induction of CEMIP in cancer was hitherto unknown. Here we demonstrate that hypoxia induces CEMIP expression leading to enhanced cell migration. Immunohistochemistry of human colon cancer tissues revealed that CEMIP is upregulated in cancer cells located at the invasive front or in the submucosa. CEMIP localization inversely correlated with E-cadherin expression, which is characteristic of the epithelial-to-mesenchymal transition. Mechanistically, hypoxia-inducible-factor-2α (HIF-2α), but not HIF-1α binds directly to the hypoxia response element within the *CEMIP* promoter region resulting in increased CEMIP expression. Functional characterization reveals that CEMIP is a downstream effector of HIF-2α-mediated cell migration. Expression of CEMIP was demonstrated to negatively correlate with the expression of Jarid1A, a histone demethylase that removes methyl groups from H3K4me3 (an activation marker for transcription), resulting in altered gene repression. Low oxygen tension inhibits the function of Jarid1A, leading to increased presence of H3K4me3 within the *CEMIP* promoter. These results provide insight into the upregulation of CEMIP within cancer and can lead to novel treatment strategies targeting this cancer cell migration-promoting gene.

## INTRODUCTION

Cancer metastasis remains a leading cause of treatment failure for cancer patients despite advancements in our understanding of this complex process. The characterization of genes involved in the initial steps of metastasis, including cell migration, could lead to novel targets for preventing cancer dissemination. We and others recently identified *KIAA1199* as a novel cancer cell migration-promoting gene, referred to now as Cell Migration Inducing Protein (CEMIP), and linked CEMIP's expression to the maintenance of a mesenchymal-like phenotype and metastatic potential [1, 2]. Clinical significance of CEMIP in cancer has been highlighted by its upregulation in numerous human cancers, including breast, gastric, and colon cancers, and its negative correlation with patient survival [1, 3–5]. Together, these studies demonstrate the vital role of CEMIP in cancer progression and warrant further investigation into the regulatory mechanism(s) of CEMIP expression in cancer. Previous analysis of the *CEMIP* promoter revealed both genetic and epigenetic regulatory mechanisms. Transcription factors AP-1 and NF-kB were both found to be required for general transcription of *CEMIP* [2, 3]. Additionally, hypomethylation of the CpG island within the promoter region was observed in aggressive cancer cell lines and in isolated human breast cancer cells [3]. Interestingly, a correlation between CEMIP expression and hypoxic stress has been observed [6], suggesting a possible link between CEMIP expression and hypoxia.

Hypoxia is one of the most common stressors encountered within the tumor microenvironment [7]. It occurs in solid tumors due to rapid tumor growth and insufficient and disorganized angiogenesis. This lack of available oxygen drives malignant progression by imposing a powerful selective pressure, resulting in a more aggressive population of cancer cells that can resist death and escape the environment [8, 9]. The cellular responses to hypoxic stress are mediated by the hypoxia-inducible-factor (HIF) heterodimer that consists of HIF-α and HIF-1β [10, 11]. HIF-1β is constitutively expressed, independent of oxygen levels within the cell, whereas HIF-α, encoded by three genes (HIF-1α, -2α and -3α), serves as the oxygen sensing subunit [12]. Under normoxia, proline residues within HIF-α are hydroxylated, targeting it for proteasomal degradation [12]. Under low oxygen conditions, HIF-α can accumulate and dimerize with HIF-1β in order to bind to the hypoxia response elements (HRE) within promoter regions and activate target genes necessary for cellular adaptation [13, 14].

In addition to the genetic alterations initiated by the HIF complex, recent evidence supports changes in epigenetic regulatory mechanisms under hypoxic stress. Various covalent modifications, including methylation of histone proteins, have an impact on the transcriptional activity of genes involved in cancer [15]. Exposure to hypoxia leads to increased expression of histone modifying enzymes and global changes in methylation patterns that result in either repression or activation of genes [16–18]. Of particular interest is the trimethylation of lysine 4 of histone H3 (H3K4me3), an activation marker for gene transcription [19], shown to be induced by hypoxic stress [20]. The increased presence of H3K4me3 in hypoxia has been shown to result from the inhibition of the demethylase activity of Jarid1A/RBP2 (retinoblastoma protein 2), which requires oxygen to function [20]. Jarid1A, a member of the JmjC-domain containing family of proteins [21], has been shown to specifically remove the methyl groups from tri- and dimethylated lysine 4 of H3 proteins resulting in decreased transcription of targeted genes [22, 23]. The effect of Jarid1A on transcriptional activity of genes involved in cancer progression has not been extensively studied.

Hypoxic stress results in a genetic reprogramming that ultimately results in a transformation of cancer cells into a more aggressive phenotype. Based on CEMIP's role in cancer cell invasiveness, we hypothesized that exposure to hypoxic conditions could lead to the upregulation of CEMIP in cancer cells resulting in cancer dissemination. In this study, we unraveled the regulatory mechanism of CEMIP expression under hypoxic conditions. Importantly, we linked hypoxia to a cascade of HIF-2α-Jarid1A-H3K4me3 to enhanced CEMIP transcription in colon cancer dissemination. Discovering the mechanism by which cancer cells specifically induce CEMIP, leading to a more aggressive phenotype, can have a positive impact on potential therapies targeting this gene.

## RESULTS

### Upregulation of CEMIP in invasive and metastasized human colon cancer cells

**Figure 1:**
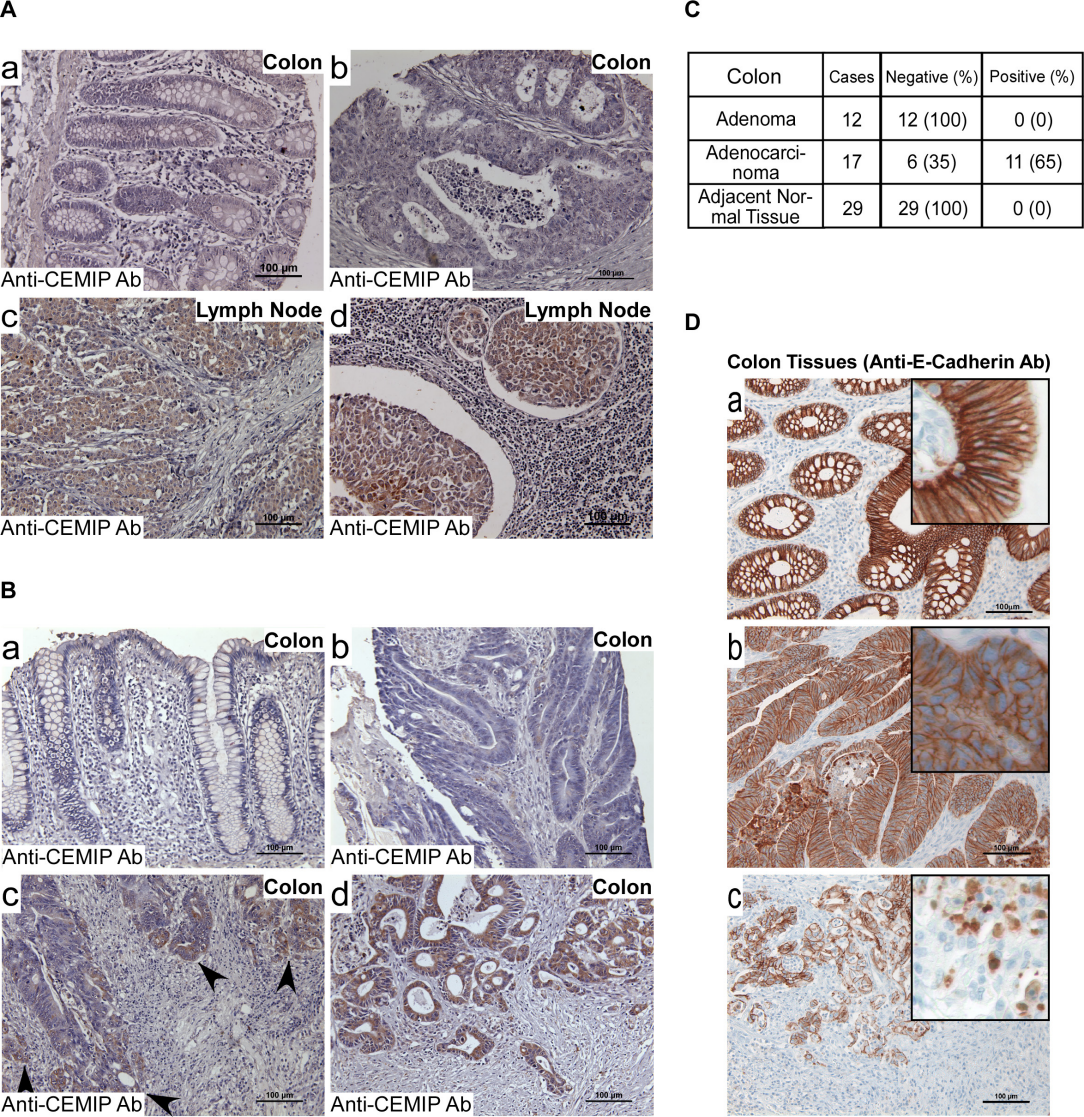
Upregulation of CEMIP in human invasive and metastatic colon cancer **A and B.** A human colon cancer tissue microarray (US Biomax CO1005) containing tumor adjacent normal tissue (A-a), adenocarcinomas (A-b), and adenocarcinomas metastasized to lymph nodes (A-c & d), and individual human colon adenocarcinoma specimens containing cancer adjacent normal tissues (B-a), primary tumor (B-b), invasive front (B-c), and invaded tumor cells in submucosa (B-d) were examined by immunohistochemistry (IHC) with an anti-CEMIP antibody. Representative images of tissue types and staining intensities of colon cancer tissue array and tissue samples are shown. Bar = 100 μm. **C.** Total number of individual specimens from human colon adenoma, adenocarcinoma and adjacent normal tissue examined by IHC for CEMIP expression. **D.** Individual human colon adenocarcinoma specimens containing cancer adjacent normal tissues (D-a), primary tumor (D-b), and invaded tumor cells in submucosa (D-c) were examined by IHC with an anti-E-cadherin antibody. Representative images are shown. Bar = 100 μm. The staining for CEMIP in panel B and E-cadherin in panel D were from the same case. Inserts represent enlarged images of representative positive cells.

With recent reports highlighting the functional significance of CEMIP in cancer cell survival, migration and invasion [1, 2], the need to ascertain the mechanism of upregulation of CEMIP in cancer cells is imperative. To study the regulatory mechanisms of CEMIP in solid tumors, we first examined the expression pattern of CEMIP in human colon cancer specimens by immunohistochemical (IHC) staining using a human colon tissue microarray (TMA) that contains 30 paired tumor and adjacent normal colon tissues and 40 lymph nodes positive for colon cancer (Supplemental Table 1). Surprisingly, 66% of primary colon cancer specimens were negligibly stained with a well-characterized anti-CEMIP antibody (Fig. 1A–1b), which is in contrast to a previous report of bulk colon cancer tissue specimens examined by DNA microarray [24]. Among the 34% of positively stained tumors, most of the cases contained colon cancer cells invading into the submucosa, suggesting CEMIP may be upregulated only in invasive colon cancer cells. In agreement with this assumption, a high incidence of positive staining of CEMIP (76%) was observed in colon cancer cells that metastasized to lymph nodes (Fig. 1A-1c, 1d). There was no staining of CEMIP in the normal colon tissue (Fig. 1A–1a). These IHC data suggest that CEMIP is associated with the invasive capability of colon cancer cells.

Since CEMIP mRNA level has been reported to be upregulated in human cancer specimens using samples from bulk tumor tissues [4, 24] and TMA samples have limited representation of primary tumors, we next examined formalin-fixed, paraffin-embedded (FFPE) individual human colon specimens using IHC. Within the primary tumor specimens, adjacent normal tissues (Fig. 1B–1a), as well as most adenocarcinoma cells in the mucosa (Fig. 1B–1b), were negligibly stained by the anti-CEMIP antibody. However, tumor cells at the invasive front and in the submucosa were positively stained for CEMIP (Fig. 1B–1c & 1d). Overall, 65% of adenocarcinoma specimens showed this staining pattern. In contrast, colon adenomas were negligibly stained (Fig. 1C). A Chi-square test with exact *p*-value suggests that adenocarcinomas have a significantly higher percentage of tissue samples presenting with CEMIP positive staining (*p*-value < 0.0001).

Initiation of cancer invasion is often associated with cellular phenotypic changes referred to as epithelial-to-mesenchymal transition (EMT). To determine if upregulated CEMIP in invaded colon cancer cells coincides with changes associated with the EMT phenotype, E-cadherin, a marker commonly lost during EMT, was examined using an anti-E-cadherin antibody. As expected, E-cadherin was strongly detected at the cell-cell junction area in cancer adjacent normal tissue (Fig. 1D–1a). Although primary tumors were also positive for E-cadherin (Fig. 1D–1b), the intensity of the E-cadherin staining was reduced as compared to adjacent normal tissue. Importantly, tumor cells located at the invasive front as well as those that had invaded into the submucosa, presented with a loss of cell-cell junction E-cadherin, suggesting EMT may have occurred in those invading cancer cells (Fig. 1D-1c). This inverse correlation between upregulated CEMIP and downregulated E-cadherin in cancer cells at the invasive front reinforces the role of CEMIP in cancer EMT [1, 2].

### Induction of CEMIP under hypoxic conditions

**Figure 2:**
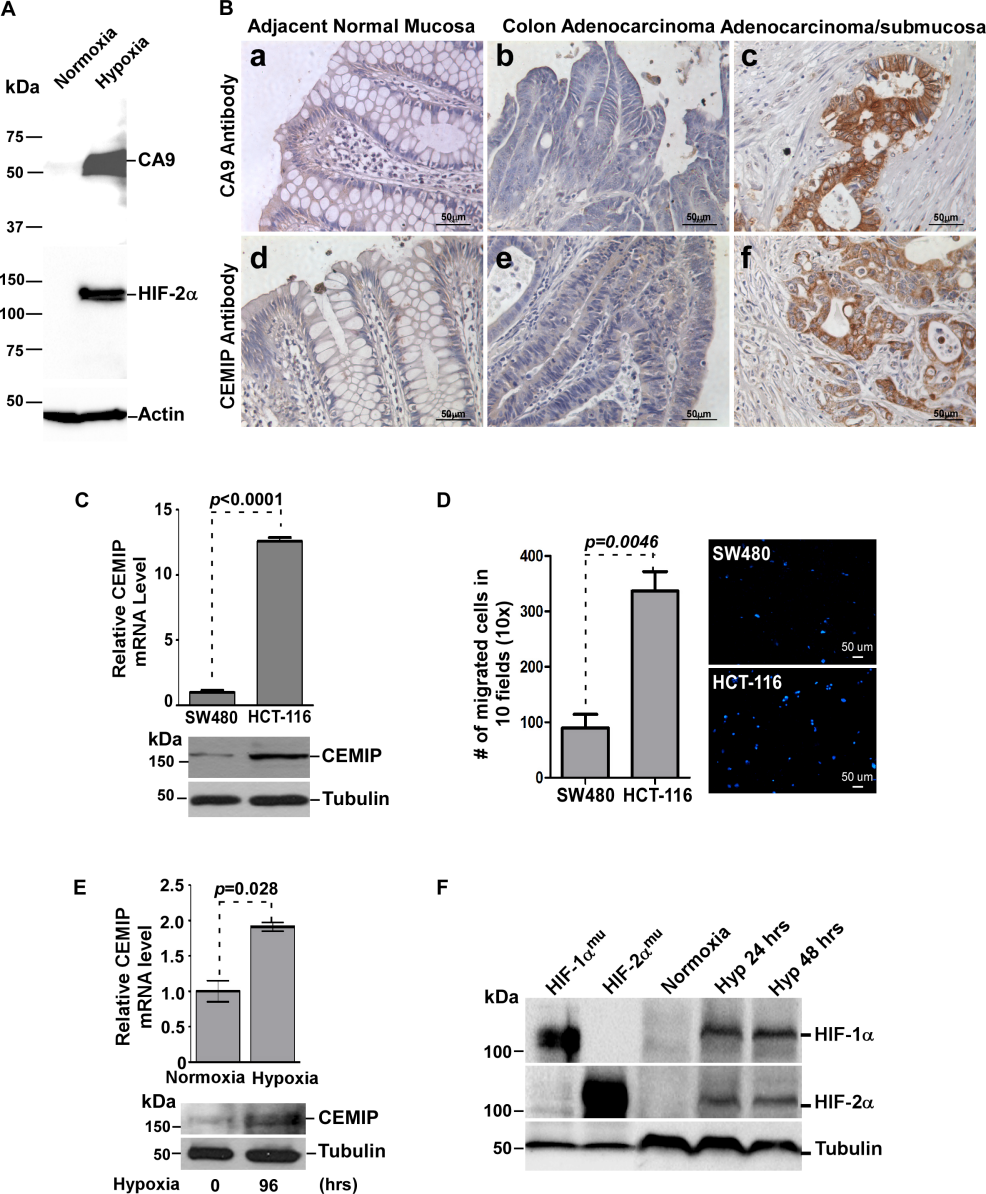
Hypoxia induces CEMIP expression in human colon cancer **A.** Hypoxia induces HIF2-α and CA9 in SW480 cells. Total cell lysates from SW480 cells cultured under either normoxia or hypoxia for 48 hours were examined by Western blotting using antibodies as indicated. Actin was used as loading control for Western blotting. **B.** Human colon adenocarcinoma specimens were stained with anti-CA9 and anti-CEMIP antibodies separately. Representative images from the same case are shown. **C.** Total RNA and lysates isolated from SW480 and HCT-116 cells were analyzed by real time RT-PCR using CEMIP-specific primers and Western blotting using anti-CEMIP antibody. The mRNA expression was normalized using housekeeping gene HPRT-1 and tubulin was used as loading control for Western blotting. **D.** Cell migratory ability of SW480 and HCT-116 cells was examined by a Transwell chamber migration assay. Migrated cells were stained by Hoechst. Representative fields are shown in the right panel. **E.** Total RNA and lysates were analyzed by real time RT-PCR and Western blotting in SW480 cells cultured under normoxic (21% O_2_) or hypoxic (1% O_2_) conditions for indicated times. **F.** Western blot analysis of whole cells lysates from MCF-7 cells cultured under normoxic or hypoxic conditions for indicated times. MCF-7 cells transfected with HIF-1α^mu^ or HIF-2α^mu^ cDNAs were used as positive controls. Tubulin was used as a loading control.

Since solid tumors are often accompanied by hypoxia, we next explored if tumor cells with upregulated CEMIP are located in a hypoxic environment. Hypoxia-induced factor-α (HIF-α) is an intrinsic marker of tissue hypoxia. However, HIF-α proteins in cells are rapidly degraded when tissue specimens are processed under normoxic conditions. Considering the degradation of HIF-α in FFPE samples, carbonic anhydrase 9 (CA9), a surrogate marker of hypoxia [25, 26], was employed to identify hypoxia-affected cells. Indeed, a positive correlation of HIF-2α expression with CA9 was observed in human colon cancer SW-480 cells cultured under hypoxic conditions (Fig. 2A). The expression of CA9 in the FFPE colon cancer specimens was then examined by IHC using the anti-CA9 antibody. Normal mucosa adjacent to colon tumors and most of the colon cancer cells near the mucosal surface were negatively stained for CA9 (Fig. 2B-2a, 2b), whereas cancer cells located at the invasive front within deep mucosa or invading into the submucosa showed strong cell surface staining for CA9 (Fig. 2B-2c). Interestingly, cell surface staining of CA9 in colon cancer cells positively correlates with cytoplasmic staining of CEMIP (Fig. 2B-2c, 2f). This observation suggests a link between hypoxia and CEMIP expression in human colon cancer.

To further determine the link between hypoxia and CEMIP expression, we carried out *in vitro* studies using two well-characterized human colon cancer cell lines, less aggressive SW480 cells and aggressive HCT-116 cells. HCT-116 cells had a significantly higher level of CEMIP expression and increased migratory ability compared to SW480 cells (Fig. 2C & 2D), suggesting a positive correlation between CEMIP expression and cell migration in colon cancer. This observation was confirmed by both loss- (using aiRNA against CEMIP) and gain (using CEMIP cDNA)-of-function studies (Supplemental Figs. 1 and 2). Asymmetric interfering RNA (aiRNA) is a novel approach for gene silencing. It enhances gene silencing efficiency and significantly reduces off-target effects [27, 28]. When SW480 cells were cultured under hypoxic conditions (1% O2) for 4 days, CEMIP mRNA and protein levels were significantly increased compared to normoxic conditions (Fig. 2E). Hypoxic conditions were verified by the induction of both HIF-1α and HIF-2α after 24 and 48 hours (Fig. 2F). Two constitutively active HIF mutants, HIF-1α^P402A/P564A^ (HIF-1α^mu^) and HIF-2α^P405A/P531A^ (HIF-2α^mu^) were used as molecular weight controls (Fig. 2F).

To determine if this response to hypoxic stress was common among other cancer cell types, the expression of CEMIP was also examined in HeLa cells, a human cervical cancer cell line, and MCF-7 cells, a non-aggressive human breast cancer cell line. A significant increase in CEMIP expression at both the mRNA and protein levels was observed in both these cell lines after a shorter exposure time to hypoxia (Supplemental Figs. 3 and 4), suggesting that upregulated CEMIP in tumor cell lines is a general consequence of hypoxic stress and not tissue specific.

### Transcriptional regulation of CEMIP by hypoxia inducible factor-2α

**Figure 3:**
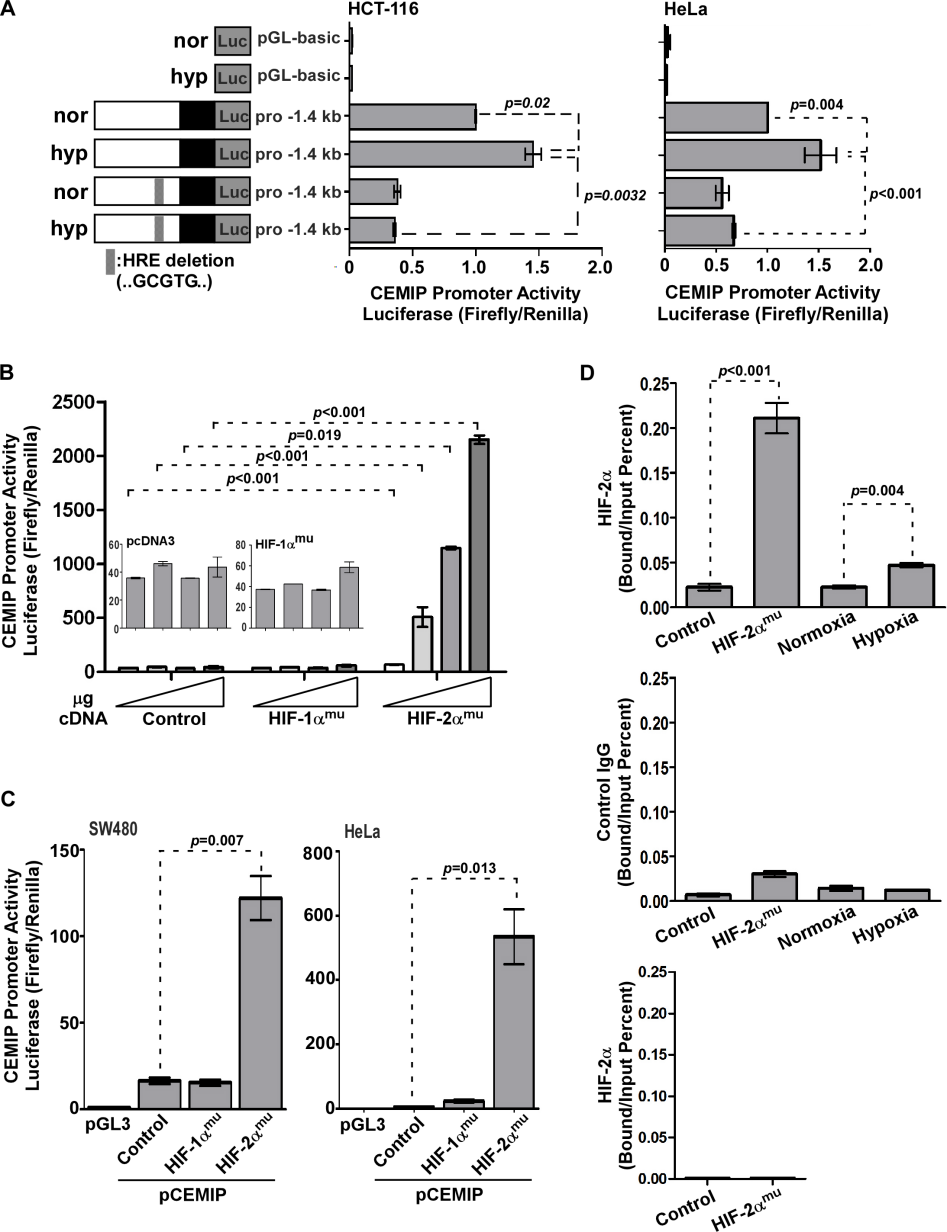
HIF-2α directly induces CEMIP transcription **A.** Dual luciferase reporter assay using lysates from HCT-116 and HeLa cells transfected with pGL3 basic (control), wild-type pro-1.4 kb *CEMIP*, or ΔHRE pro-1.4 kb *CEMIP* promoter-luciferase reporter cDNAs cultured under normoxia (nor) or hypoxia (hyp) for 48 hours. Renilla luciferase was used as a normalization control. **B.** Dual luciferase reporter assay using lysates from COS-1 cells transfected with *CEMIP* promoter-luciferase reporter cDNA along with increasing amounts of vector control or HIF-α mutant cDNAs as indicated. Renilla luciferase was used as a normalization control. **C.** Dual luciferase reporter assay using lysates from SW480 and HeLa cells co-transfected with indicated cDNAs. Renilla luciferase was used as a normalization control. **D.** A chromatin immunoprecipitation (ChIP) assay using HeLa cells either transfected with indicated cDNAs or cultured under normoxia or hypoxia for 48 hours. An anti-HIF-2α-specific antibody or an IgG control antibody was used for the ChIP. Primers spanning the HRE within the *CEMIP* promoter region were used for quantitative PCR. Primers that span the outside of the hypoxia response element within the CEMIP promoter were used as negative control.

To determine if hypoxia transcriptionally induces CEMIP expression, we analyzed the 5′ flanking region of *CEMIP* for potential hypoxia response element(s) (HREs). Bioinformatic analysis of the *CEMIP* promoter revealed a potential HRE spanning from −125 bp to −120 bp (GCGTG) in the sense orientation, suggesting that the upregulation of CEMIP could be due to enhanced promoter activity. To determine if the deduced HRE site within the promoter of *CEMIP* is a functional HRE that regulates CEMIP expression under hypoxia, a reporter plasmid with the predicted HRE site deleted was generated. HCT-116 cells transfected with cDNAs as indicated were cultured under either normoxic or hypoxic conditions followed by a luciferase reporter assay [3]. Exposure to hypoxic stress significantly increased *CEMIP* promoter activity as compared to basal levels seen under normoxia in HCT-116 cells (Fig. 3A). However, hypoxia failed to induce the activity of the ΔHRE *CEMIP* promoter to the same level observed in cells expressing the wild-type promoter (Fig. 3A). This observation is also reproduced in HeLa cells (Fig. 3A). We and others have recently reported that transcription factors AP-1 and NF-kB also contribute to *CEMIP* promoter activity [2, 3], which could explain why complete inhibition of luciferase activity was not seen upon HRE deletion. Together, these results provide evidence to suggest that hypoxia directly induces the transcription of *CEMIP*.

Since both HIF-1α and -2α were stabilized under hypoxia, we next examined which HIF-α isoform is specifically required for hypoxia-induced CEMIP expression. HIF-1α^mu^ and HIF-2α^mu^, which are resistant to hydroxylation under normoxic conditions (Figure 2F) [29, 30], were employed. The reporter gene assay was performed in a non-tumor COS-1 cell line because a more direct effect can be obtained from a transient transfection. Co-transfection of COS-1 cells with the wild-type *CEMIP* promoter plasmid and the HIF-2α^mu^ cDNA resulted in an increase in *CEMIP* promoter activity (Fig. 3B). No significant induction of promoter activity was observed in cells expressing either vector or HIF-1α^mu^ even with increasing amounts of cDNA used for transfection, further supporting the specific induction of *CEMIP* via HIF-2α. This conclusion is not cell type specific, demonstrated by the significantly increased *CEMIP* promoter activity upon overexpression of HIF-2α^mu^ but not HIF-1α^mu^ or vector cDNA in SW480 and HeLa cells (Fig. 3C).

To investigate whether HIF-2α binds directly to the HRE site within the *CEMIP* promoter, a chromatin immunoprecipitation (ChIP) assay coupled with quantitative PCR was performed. Overexpression of HIF-2α^mu^ in HeLa cells caused a significant increase in HRE occupancy at the proximal *CEMIP* promoter compared to vector control cells (Fig. 3D). This observation was further confirmed in HeLa cells cultured under hypoxic conditions (Fig. 3D). There was negligible amplification when normal control IgG or primers that span the region outside of the hypoxia response element within the CEMIP promoter were used (Figure 3D bottom panels). Our data demonstrate that hypoxia-upregulated CEMIP expression is partially driven by HIF-2α.

### Requirement of CEMIP in HIF-2α-mediated cell migration

**Figure 4:**
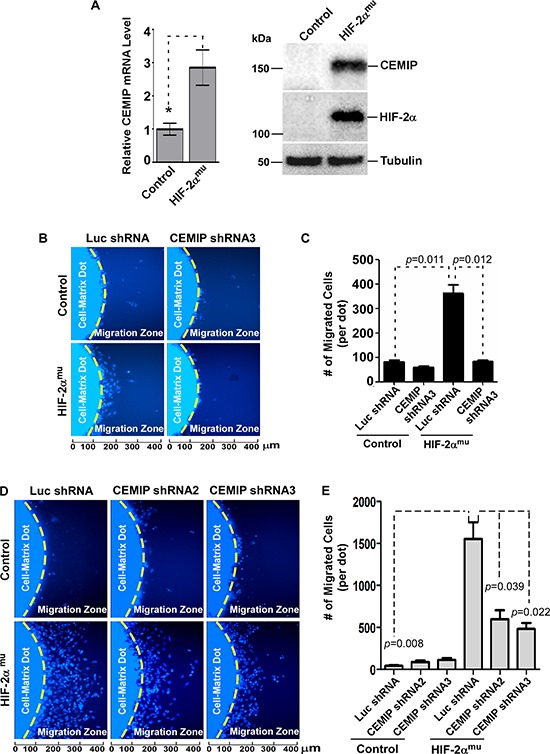
HIF-2α leads to increased cell migration that is dependent on upregulated CEMIP **A.** Left Panel: Real time RT-PCR analysis of CEMIP mRNA expression in SW480 cells transfected with vector (control) or HIF-2α^mu^ cDNA. The expression of CEMIP was normalized using housekeeping gene HPRT-1. Right Panel: Western blot analysis of whole cells lysates from SW480 cells transfected with vector (control) or HIF-2α^mu^ cDNA. Tubulin was used as a loading control. **B & C.** 2-Dimensional (2-D) dot migration assay was performed in luciferase (Luc) shRNA control and CEMIP shRNA expressing SW480 cells transfected with either vector control or HIF-2α^mu^ cDNA. The number of migrated cells surrounding the initial cell-matrix dot (Migration Zone) was counted using Nikon NIS Elements imaging software based on nuclear Hoechst staining. Each condition was repeated three times. Representative images are shown (B) Quantification of number of migrated cells per dot is shown (C) **D & E.** 2-D migration assay performed in HeLa cells as described in B & C.

To test whether the effect of HIF-2α on the promoter activity of *CEMIP* was sufficient for an increase in endogenous CEMIP expression, CEMIP mRNA levels were measured using real-time RT-PCR and protein expression was analyzed via Western blotting of whole cell lysates following overexpression of vector control or HIF-2α^mu^ cDNA. Expression of HIF-2α^mu^ caused a significant increase in CEMIP mRNA and protein expression in SW480 cells (Figure 4A). These results were confirmed in both HeLa and MCF-7 cells (Supplemental Figs. 5 & 6).

It has been shown that stabilized HIF-2α under hypoxia promotes an aggressive phenotype in cancer cells, including increased migration, via upregulation of a variety of target genes [31, 32]. Therefore we sought to determine if HIF-2α-induced cell migration requires upregulated CEMIP. SW480 cells were infected with retrovirus encoding previously validated CEMIP shRNAs and luciferase (Luc) control shRNA as previously employed [1] in order to silence CEMIP upregulated by HIF-2α. HIF-2α^mu^ was then transiently transfected into these cells stably expressing CEMIP shRNA or Luc shRNA and a 2-dimensional cell migration assay was performed. Indeed, we observed that less invasive SW480 cells displayed enhanced cell migratory ability when mutant HIF-2α (HIF-2α^mu^ + Luc shRNA) was expressed in the cells as compared to control cells (Control + Luc shRNA) (Figs. 4B & 4C). When CEMIP was silenced, HIF-2α-mediated cell migration was significantly reduced (Figs. 4B & 4C), suggesting HIF-2α-mediated cell migration is via upregulated CEMIP.

To strengthen support for the role of HIF- 2α-upregulated CEMIP in cell migration, HeLa cells, which respond to HIF-2α by inducing CEMIP upregulation (Supplemental Fig. 5), were also employed using two shRNAs targeting CEMIP [1]. Expression of HIF-2α^mu^ in Luc shRNA control cells significantly induced cell migration (Figs. 4D & 4E). In contrast, silencing of CEMIP in HeLa cells significantly attenuated the expected HIF-2α-induced increase in cell migration (Figs 4D & 4E). Together, these data suggest a cascade of hypoxia-HIF-2α-CEMIP upregulation in enhancing cell migration as an escape mechanism for cancer cells to migrate away from hypoxic areas.

### Regulation of CEMIP expression by epigenetic changes under hypoxia

**Figure 5:**
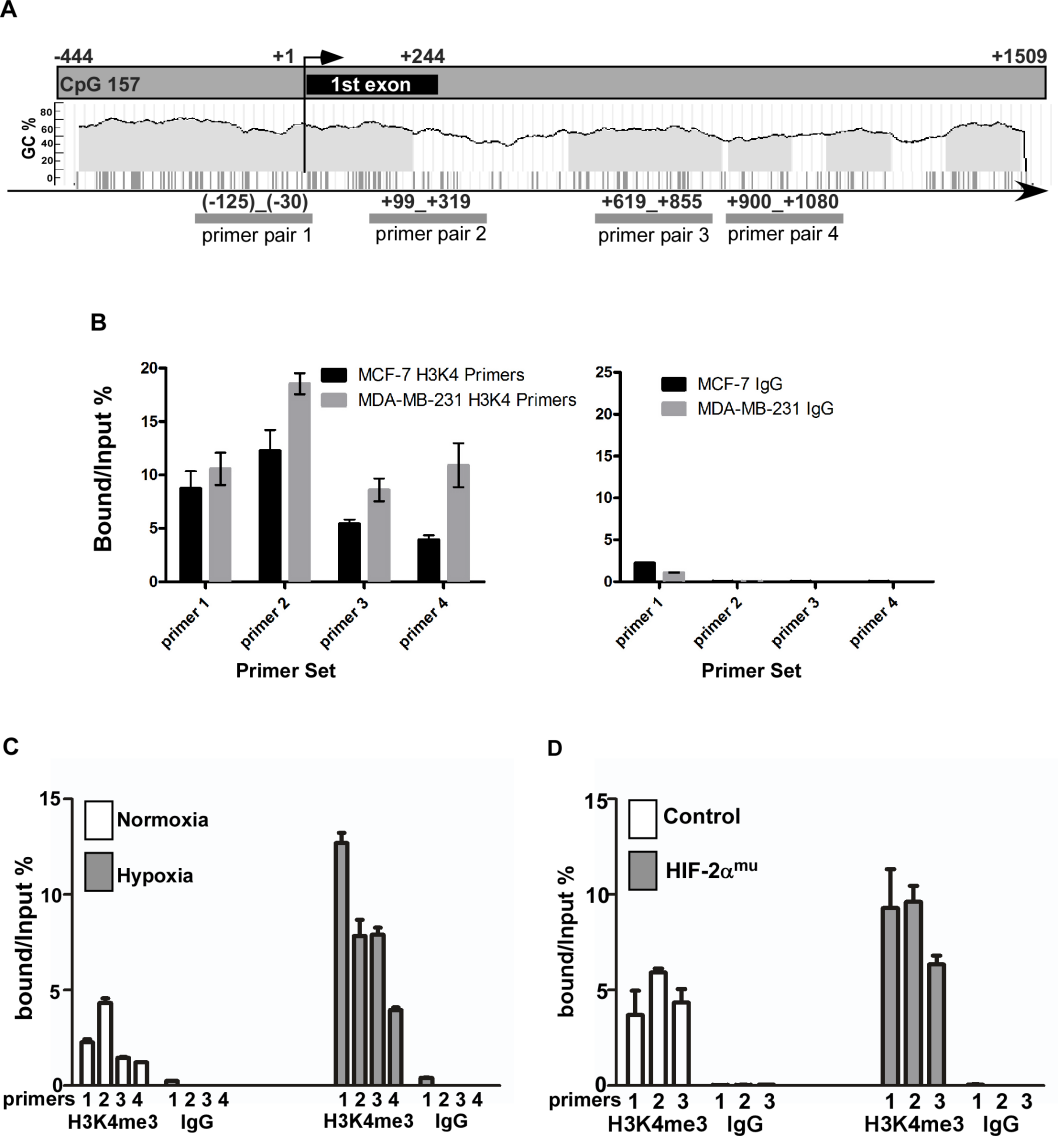
Epigenetic alterations within the *CEMIP* promoter region induced by hypoxic stress **A.** Schematic diagram of the *CEMIP* promoter region including the first exon. The locations of the primer pairs used for the ChIP experiments are indicated. **B.** A ChIP assay followed by quantitative PCR was performed using MCF-7 and MDA-MB-231 cells to assess the level of trimethylated lysine 4 of the histone 3 protein (H3K4me3) within the region indicated in panel A. An anti-H3K4me3 antibody or an IgG control antibody was used for the ChiP. **C.** A ChIP assay followed by quantitative PCR was performed using SW480 cells cultured under normoxic or hypoxic conditions for 4 days. **D.** A ChIP assay followed by quantitative PCR was performed using SW480 cells transfected with vector control or HIF-2α^mu^ cDNA.

In addition to the effects orchestrated by the HIF proteins, it has recently been demonstrated that exposure to hypoxic stress causes epigenetic alterations, such as changes in the methylation patterns of histone proteins [17]. To investigate the role of methylation marks in the regulation of *CEMIP*, a ChIP assay using an antibody that recognizes the transcription activation mark H3K4me3 [19], followed by quantitative PCR utilizing primers that flank 4 different regions within the *CEMIP* promoter and first intron (Fig. 5A) was employed. The H3K4me3 modification was highly enriched in MDA-MB-231 cells, an aggressive human breast cancer cell line that expresses high levels of CEMIP, as compared to MCF-7 cells, which express lower levels of CEMIP (Fig. 5B) [1], demonstrating a correlation between CEMIP expression and the level of the H3K4me3 mark. To ascertain if hypoxic stress leads to changes in the level of H3K4me3 within *CEMIP*, SW480 cells were cultured under normoxic or hypoxic conditions for 4 days followed by ChIP and qPCR. An enrichment of H3K4me3 at all 4 sites was observed in SW480 cells upon exposure to hypoxia (Fig. 5C). Furthermore, overexpression of HIF-2α in SW480 cells (Fig. 4A) resulted in increased levels of H3K4me3 as compared to cells expressing vector control (Fig. 5D). Together, these results suggests that under normoxic conditions, the H3K4me3 activation mark is maintained at a low level within the *CEMIP* promoter region in less aggressive cancer cell lines, therefore leading to lower expression levels of CEMIP. Upon exposure to hypoxia, the H3K4me3 levels increase leading to increased induction of *CEMIP*.

**Figure 6:**
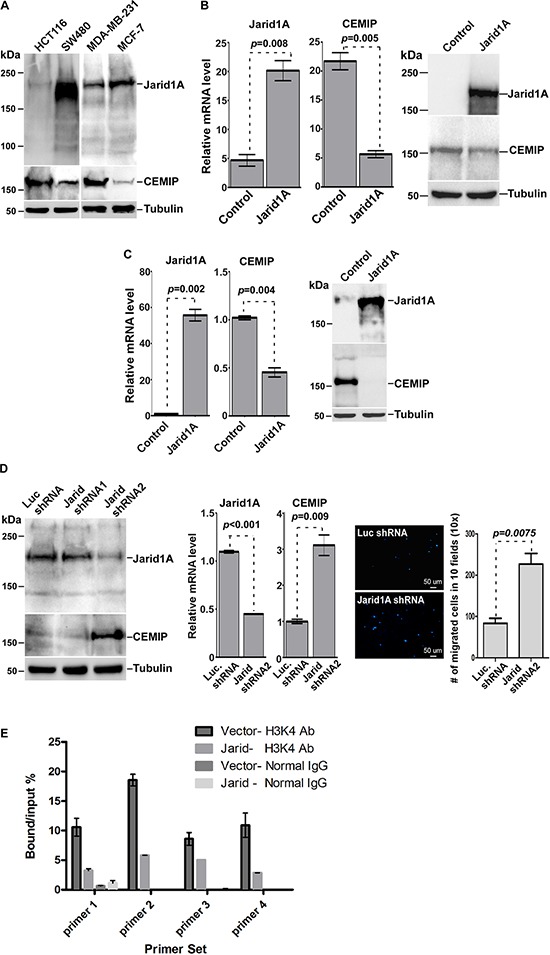
Jarid1A expression inversely correlates with CEMIP expression **A.** Western blot analysis of whole cell lysates from cells as indicated using antibodies directed against CEMIP and Jarid1A. Tubulin was used as a loading control. **B & C.** Real Time RT-PCR and Western blot analysis of HCT-116 (B) and MDA-MB-231 (C) cells for ectopically expressed Jarid1A and endogenous CEMIP. **D.** Western blot analysis was used to determine the silencing efficiency of Jarid1A shRNAs in SW480 cells (left panel). Real Time RT-PCR using the most effective Jarid1A shRNA2 was performed to monitor the effect of Jarid1A on CEMIP expression (middle panel). Transwell chamber migration assay was performed in SW480 cells expressing Jarid1A shRNA2 or control shRNA (right panel). After staining with DAPI for nuclear, the migrated cells were counted in 10 different fields using a Nikon 10x lens. **E.** Decreased levels of H3K4me3 within the *CEMIP* promoter region in MDA-MB-231 cells overexpressing Jarid1A. A ChIP assay followed by quantitative PCR was performed in MDA-MB-231 cells trasnfected with Jarid1A cDNA or vector control. An anti-H3K4me3 antibody or an IgG control antibody was used for the ChiP.

Since elevated levels of H3K4me3 in hypoxic cells has been specifically linked to inhibition of Jarid1A's demethylase activity [20], we hypothesized that Jarid1A is responsible for the low expression level of CEMIP in less aggressive cancer cell lines under normoxia due to removal of the H3K4me3 activation mark. Upon exposure to hypoxia, Jarid1A's inactivity could therefore lead to the increased presence of H3K4me3 within the *CEMIP* promoter region. To determine the role of Jarid1A in the regulation of CEMIP expression, we first surveyed cancer cell lines for expression of Jarid1A and CEMIP. Western blotting analysis revealed an inverse correlation between Jarid1A and CEMIP in cancer cell lines. High expression of CEMIP accompanied by low expression of Jarid1A was found in more invasive HCT-116 and MDA-MB-231 cells as compared to less invasive SW480 and MCF-7 cells, respectively (Fig. 6A). To further confirm the negative correlation between Jarid1A and CEMIP, a gain-of-function study of Jarid1A was performed. Overexpression of Jarid1A in both HCT-116 and MDA-MB-231 cells led to decreased CEMIP mRNA and protein levels (Fig. 6B and 6C). In contrast, silencing Jarid1A in low CEMIP expressing cell lines, e.g. SW480, resulted in an increase of CEMIP expression (Fig. 6D). There is a significant increase in cell migration upon silencing of Jarid1A in SW480 cells (Fig. 6D). Additionally, overexpression of Jarid1A in MDA-MB-231 cells resulted in decreased levels of H3K4me3 within the *CEMIP* promoter region as compared to vector control cells (Fig. 6E), further supporting the role of Jarid1A in maintaining low expression levels of CEMIP by actively removing the H3K4me3 activation mark.

## DISCUSSION

The present study has unraveled a novel cascade of the regulatory mechanism of CEMIP in human cancer progression. The key mechanism that we elucidate here to account for upregulated CEMIP in human colon cancer dissemination is that hypoxia stabilizes HIF-2α, which can then bind to the *CEMIP* promoter, and inhibit the demethylase activity of Jarid1A, which leads to increased presence of H3K4me3 within the *CEMIP* promoter, ultimately resulting in increased CEMIP expression. This conclusion is supported by a positive correlation between CEMIP expression and the hypoxia marker CA9 in human colon cancer cells as well as several lines of *in vitro* biochemical and biological studies. Characterization of the regulatory mechanism of CEMIP is significant because upregulated CEMIP is only detected in colon cancer cells located at the invasive front or submucosa examined by IHC. Moreover, requirement of upregulated CEMIP in HIF-2α-mediated cell migration further highlights the importance of CEMIP in cancer dissemination. Our data support CEMIP as a novel cancer invasion-promoting gene that is induced upon exposure of cancer cells to hypoxic conditions as part of the reprogramming associated with increased migratory capacity.

Upregulated CEMIP has been linked with decreased survival probability in patients with colon cancer and other type of cancers [1, 4, 5], suggesting that CEMIP may be used as a prognostic marker. However, there is controversy regarding the frequency of CEMIP upregulation in primary colon cancer. A previous report, utilizing DNA microarray, demonstrated that 95% of 511 colon adenocarcinoma tissues displayed upregulated CEMIP [24]. Our study revealed that only 34% of TMA from colon cancer patients were positively stained for CEMIP. When individual human colon cancer specimens were examined in detail, we observed that most of the colon cancer cells located in the mucosal layer displayed minimal expression of CEMIP. In contrast, strong positive staining of CEMIP was found in cancer cells located at the invasive front or cells invading into the submucosa layer. Our study links upregulated CEMIP expression with colon cancer dissemination. Interestingly, increased CEMIP expression in colon cancer cells correlated with the loss/relocation of E-cadherin at cell-cell adheren junctions. This observation is in agreement with previous observations that CEMIP plays a critical role in conversion of less invasive cancer cells to invasive ones via EMT [1, 2].

However, CEMIP has been reported to play roles in cancer cell proliferation and invasion either negatively or positively [1, 2, 24, 33–35]. Tiwari et. al, [35] reported that SW480 cells overexpressing KIAA1199 (CEMIP) displayed reduced cell invasive ability compared to control cells, but did not display altered cell migratory ability. Given the consideration that proteolytic activity and cell migratory ability are two key requirements for cancer invasion, Tiwari's observation suggests CEMIP may reduce proteolytic activity of cancer cells, therefore inhibiting cell invasion. In contrast, several other recent reports including the observation presented here support CEMIP's role in enhancing cancer cell migration, hence contributing to increased cancer cell invasion through different signaling pathways [1, 2, 33]. Based on clinical studies showing that expression of CEMIP is inversely correlated with cancer survival rate in patients with breast cancer, colon cancer, and gastric cancer [1, 4, 5], CEMIP could positively affect cancer cell invasion through enhanced cell migration. In addition, Tiwari et. al, [35] also reported that overexpression of CEMIP reduced SW480 cell proliferation. In contrast, other reports demonstrated that CEMIP is positively involved in cell survival pathways [1, 33, 34]. This discrepancy could be due to experimental models, cell lines, and ectopic expression versus endogenous expression.

The key mechanistic finding in our study is that hypoxia promotes CEMIP expression in invasive cancer cells. This conclusion is supported by our IHC staining in colon cancer tissues based on a well-characterized hypoxia surrogate marker, CA9 [25, 26]. Due to rapid hydroxylation of HIF-1α or -2α under normoxic conditions, it has been challenging to identify effective markers of hypoxia among HIF-α targets. CA9 expression shows colocalization with pimonidazole, a chemical marker of hypoxia, and its expression is usually restricted to perinecrotic areas [36]. Upregulated CA9 has been found at the invasive front of gastric cancers [37] that is in agreement with our observation in human colon cancer. CA9 has been reported to be regulated by HIF-1α, but not HIF-2α [38]. However, a later study demonstrated that a highly significant association of HIF-2α and CA9 was found in poorly differentiated head and neck squamous cell carcinomas [26]. Nevertheless, CA9 has been recognized as an intrinsic surrogate marker of hypoxia.

Our study links HIF-2α accumulation under hypoxia to upregulated CEMIP expression. Since HIF-1α and -2α are closely related and share a consensus binding sequence on target genes [39], it remains to be characterized as to why HIF-2α plays more of a role in the regulation of *CEMIP* transcription than HIF-1α in the cell lines tested. In a genome-wide ChIP analysis, approximately 45% and 65% of the sequences bound by HIF-1α and HIF-2α, respectively, contained the HRE consensus motif [14]. Furthermore, both *Hif-1α* and *Hif-2α* knockout mice exhibit embryonic lethality [40, 41], indicating non-redundant roles during development and possibly in different types of cancer. Interestingly, HIF-2α has previously been shown to be responsible for activating genes involved in the transition to a more aggressive phenotype, including genes involved in invasion [42], further supporting our findings. On the other hand, many target genes were reported to bind both isoforms [14]. These conflicting data further demonstrate the complexity of the transcriptional reprogramming that occurs in response to hypoxic stress. Our results showing that constitutively active HIF-2α bound to and activated the *CEMIP* promoter suggest that cooperation between HIF-2α and other transcription factors bound at the native *CEMIP* promoter may promote selective recruitment of HIF-2α to this site. A better understanding of these processes might elucidate the basic biology of how specific responses to hypoxia are coordinated, and how pharmacological manipulation of hypoxia-related pathways might be developed to interfere with CEMIP expression in human cancer.

The recent discoveries that hypoxic stress also influences the histone code lead us to inquire about the potential epigenetic mechanisms involved in the regulation of CEMIP. The result in Fig. 5B reveals the presence of the H3K4me3 activation mark in both MCF-7 and MDA-MB-231 with the latter having increased enrichment at the sites tested. The lack of a complete absence from MCF-7 cells is not surprising since there is some basal level of transcription of CEMIP in these cells. It would be interesting to examine the methylation patterns of cancer cells versus normal tissue to determine if there is a complete lack of methylation within the *CEMIP* promoter in noncancerous tissue, which would further explain the upregulation of CEMIP. In addition, it has recently been demonstrated that HIF preferentially binds to promoters that are active or at least poised for activation upon stimulation, which was characterized by either the presence of the H3K4me3 modification or RNA Polymerase II occupancy [43]. The increase in H3K4me3, due to the decreased function of Jarid1A, in MCF-7 and SW480 cells upon exposure to hypoxia could allow for HIF binding to the HRE within the *CEMIP* promoter. Together, our data reveals a regulatory cascade through which hypoxic stress leads to upregulated CEMIP in cancer cells.

The data presented strongly supports the role of Jarid1A as the demethylase that is responsible for the difference in levels of H3K4me3 in the various cell types tested. Jarid1A overexpression was shown to decrease CEMIP levels, while knocking down Jarid1A, which would be phenotypically similar to inhibiting its activity via lack of oxygen, significantly elevated CEMIP. Our studies did not rule out the possibility of other demethylases or identify the potential methyltransferases that initially mark the *CEMIP* promoter for activation. There have been studies that demonstrate crosstalk between methyltransferases and demethylases, like Jarid1A, that ultimately result in changes in transcriptional activity [44]. The histone modifications within *CEMIP* induced by hypoxic inhibition of Jarid1A can ultimately affect expression of CEMIP by keeping CEMIP in a poised state for HIF-2α or other transcription factor binding. Overall, characterizing the effect of Jarid1A on *CEMIP* is the first step towards gaining a better understanding of the epigenetic regulatory mechanisms governing the expression of CEMIP in cancer.

Based on the data present and the fact that solid tumors are often accompanied by hypoxia, which creates a selective pressure that allows more apoptosis resistant and aggressive tumor cells to survive, targeting key regulators of hypoxia, such as HIF-alpha, is a viable approach to prevent cancer dissemination. However, in the past several years, anti-angiogenic therapies have been the focus of anti-cancer strategies, but this often leads to hypoxic environments that foster malignant progression. Therefore, combining drugs that would decrease the blood supply, ultimately starving the cancer cell, while also inhibiting the activation of pathways that allow cells to resist death or gain migratory ability, has been suggested as a better approach [45]. Since CEMIP is regulated by HIF-2α, targeting HIF-2α may inhibit cancer cell migration hence blocking cancer cell invasion. It is possible to develop reagents inhibiting CEMIP expression; however, since CEMIP has been reported to be expressed in the central nervous system [46], targeting CEMIP directly may cause side effects. More fine-tuned studies to elucidate the role of CEMIP in maintaining the integrity of the central nervous system, as well as to fully characterize the regulation of CEMIP under both normal and pathological conditions can potentially lead to novel treatment strategies aimed at inhibiting the upregulation of this cell migration-promoting gene.

## MATERIALS AND METHODS

### Materials

Oligo primers were synthesized by Operon. The anti-KIAA1199 (CEMIP) antibody was purchased from Abcam. The anti-HIF-1α antibody was purchased from BD Transduction Laboratories and the anti-HIF-2α antibody was purchased from Novus Biologicals. The anti-α/β-tubulin, -E-cadherin, and -Jarid1A antibodies were purchased from Cell Signaling Technology. HIF-1α P402A/P564A (plasmid 18955), HIF-2α P405A/P531A (plasmid 18956), and Jarid1A plasmids (plasmid 14800) were purchased from Addgene. Dual Glo-Luciferase assay was purchased from Promega. aiRNA against CEMIP was purchased from Boston Biomedical, Inc. [27].

### Cell Lines, treatment of cells, and transfection

All cell lines used in this study, except GP2-293 cell line, were purchased from American Type Culture Collection (ATCC, Manassas, VA) and cultured as recommended. The GP2–293 cell line used for retrovirus production was purchased from Clontech (Mountain View, CA). Both ATCC and Clontech authenticate human cell lines in their collections. Reauthentication was not performed since the early passages of each cell line from ATCC and Clontech were used in this study. For transient transfection of cells, polyethyleneimine (MW: 250 K, Polysciences) was incubated with plasmid DNA for 30 min at room temperature before adding to cells. Medium was replaced after 18 hrs. Assays were performed after 48 hrs. For hypoxic conditions, cells were incubated using a BioSpherix ProOx C21 CO_2_ and O_2_ controller set to 1% O_2_ and 5% CO_2_.

### DNA constructions

The constructions of the 1.4 kb *CEMIP* promoter luciferase reporter, Myc-tagged CEMIP cDNA, and shRNA for CEMIP and control luciferase were previously described [1, 3]. Deletion of the HRE binding site within the *CEMIP* promoter was carried out using a site-directed mutagenesis kit (Agilent Technologies) with the 1.4 kb *CEMIP* promoter serving as the template. Small interfering oligonucleotides specific for human Jarid1A were designed using Block-iT RNAi Designer (Invitrogen) for mammalian RNA interference. The targeted regions for Jarid1A (NM_001042603.2) are as follow: Jarid1A shRNA1: GCAGCAATTACGCTTATTTGG (nt: 3405–3425), Jarid1A shRNA2: GCCTCCATTTGCCTGTGAAGT (nt: 538–558), and Jarid1A shRNA3: GCAAGATTGTTGCCAGCAAAG (nt: 726–746). A sequence of GCTTCCTGTCAC derived from miR-23 [47] was used as a shRNA loop. Oligos were annealed and cloned into the RNAi-Ready pSIREN-Retro Q vector (Clontech) at BamHI and EcoRI sites. The accuracy of all the constructs was confirmed by DNA sequencing.

### Chromatin immunoprecipitation (ChIP)

The ChIP assay was performed based on the Abcam X-ChIP (cross-linked) protocol using either the anti-HIF-2α antibody or the anti-H3K4me3 antibody. Briefly, cellular proteins were cross-linked with chromosomal DNA by 0.75% formaldehyde followed by sonication. Lysates containing 25 μg DNA were immunoprecipitated with indicated antibody or rabbit IgG as a control (Millipore) at 4°C overnight in the presence of protein-A agarose beads. Immunoprecipitated DNA was amplified by quantitative real-time PCR using either a pair of primers spanning the HRE site within the *CEMIP* promoter (Forward: 5′ GCGTGGAGGGAAGTTTCAT; Reverse: 5′ AGGCCGCTTTTATAGCCACT) or primers spanning various regions indicated in Fig. 5A (Primer pair 1: Forward 5′ GCGTGGAGGGAAGTTTCAT; Reverse 5′ AGGCCGCTTTTATAGCCACT. Primer pair 2: Forward 5′ CTGAACCCAGATTTCCCAGA; Reverse 5′ GCCCCCACTTCTTACCTCTC. Primer pair 3: Forward 5′ CGAGGAGGGAGTACCACAAG; Reverse 5′ CCCGGGTTCATCTAACATTG. Primer pair 4: Forward 5′ TGGAAGAAGGTCTGGTGGTC; Reverse 5′ CTCTCATGAGCACACGCATC). For the negative control, primers spanning the outside of the hypoxia response element within the CEMIP promoter were designed as forward primer (5′ GAGATCCTGAGACTTAGCC 3′) and reverse primer (5′ GGAGACGCAGACCTGAGC 3′). Immunoprecipitated DNA was calculated according to the bound (immunoprecipitated chromatin)/input ratio.

### Immunohistochemistry

Formalin-fixed, paraffin-embedded (FFPE) tissue sections (5 μm) of human adult colon containing either benign or adenocarcinoma tissues, as well as the human colon (CO1005) tissue array from US Biomax Inc., were examined using a standard immunohistochemistry method. Antigen retrieval was achieved by boiling tissue sections for 30 minutes in 0.01M sodium citrate, pH 4. Endogenous peroxidase was quenched by 3% H_2_O_2_. Sections were then blocked for one hour in 1% BSA at room temperature and incubated in anti-CEMIP antibody, anti-E-cadherin antibody, or anti-CA9 antibody [48] at 1:300 dilution at 4°C overnight. After washing, samples were incubated with HRP-conjugated anti-rabbit IgG at 1:200 dilution, and then by Biotin-XX-Tyramide amplification (Invitrogen, Carlsbad, CA), performed according to manufacturer's instructions, and streptavidin-HRP at 1:200 dilution. All antibodies and streptavidin-HRP were diluted in 1% BSA, and sections were washed 4 times in TBS between staining steps. Stained sections were visualized using 3, 3′-diaminebenzidine tetrahydrochloride (DAB) and counterstained with hematoxylin.

### Quantitative real-time PCR

RNA from cultured cells was isolated using Qiagen RNeasy Kit according to the manufacturer's instructions. Reverse transcriptase (BioRad iScript cDNA Synthesis Kit) was used to generate cDNA. Quantitative real-time PCR was performed using BioRad iQ SYBR-Green Super Mix on a BioRad iQ5 Real Time PCR machine. Relative expression was calculated using the ΔΔCt method. HPRT-1 was used as an internal control. Primers used for detection of CEMIP were Forward: 5′ GCTCTTGAGTTGCATGGACA and Reverse: 5′ ACCGCGTTCAAATACTGGAC. Primers used for detection of Jarid1A were Forward: CAACGGAAAGGCACTCTCTC and Reverse: CAAAGGCTTCTCGAGGTTTG.

### Dual glo-luciferase assay

Cells were transiently co-transfected with the *CEMIP* promoter luciferase reporter cDNA and the Renilla reporter cDNA using polyethyleneimine. Forty-eight hours after transfection the cells were lysed using 1x Passive Lysis Buffer and lysates were used in the Dual-Glo Luciferase Assay System according to the manufacturer's protocol. For cells cultured under normoxia or hypoxia, cells were co-transfected with the *CEMIP* promoter luciferase reporter cDNA and the Renilla reporter cDNA. Twenty-four hours after transfection the cells were cultured for 2 days under indicated conditions, lysed, and analyzed.

### 2-D dot migration assay

A collagen-cell mixture was dotted in a 96-well plate in a similar fashion to the 3-D invasion assay. Following collagen solidification, cell-matrix dots were overlaid with complete media. Cells were allowed to migrate for up to 8 hours. Cells were then stained in Hoechst/PBS (1:2000) and images were captured using the previously described microscope and camera system. Migration was then quantified by counting nuclei using the Nikon Elements Basic Research Software analysis tools.

### Transwell chamber migration assay

Polycarbonate membranes with 8 μm pore size were assembled in Blind-Well Chemotactic Chambers (Neuro Probe, MD). The lower chamber was filled with DMEM containing 10% FBS. The upper chamber was filled with 20,000 cells suspended in the same FBS containing media. Chambers were incubated for 18 h at 37 00BA;C. The cells remaining on the top surface of the membrane were removed with application of a cotton swab followed by three PBS washes. The cells on the bottom surface of the membrane were fixed and stained in Hoechst/PBS (1:2000) for 20 minutes and imaged using a Nikon Eclipse TE2000-S equipped with a Sutter Instruments SmartShutter System and a QiClick QImaging camera. Migrated cells were counted with the assistance of the Nikon Elements Basic Research Software analysis tools.

### Statistics

Student's unpaired two sided *t*-test was used to assess differences with *p* values < 0.05 specifically stated in figures and *p* < *0.001 denoted as* ***. Pooled data were presented as mean ± SD. Cell migration studies were presented as mean ± SEM for combined data of three independent experiments. All experiments were repeated at least three times.

## SUPPLEMENTARY FIGURES AND TABLE





## References

[1] Evensen NA, Kuscu C, Nguyen HL, Zarrabi K, Dufour A, Kadam P, Hu YJ, Pulkoski-Gross A, Bahou WF, Zucker S, Cao J (2013). Unraveling the role of KIAA1199, a novel endoplasmic reticulum protein, in cancer cell migration. J Natl Cancer Inst.

[2] Shostak K, Zhang X, Hubert P, Goktuna SI, Jiang Z, Klevernic I, Hildebrand J, Roncarati P, Hennuy B, Ladang A, Somja J, Gothot A, Close P, Delvenne P, Chariot A (2014). NF-kappaB-induced KIAA1199 promotes survival through EGFR signalling. Nature communications.

[3] Kuscu C, Evensen N, Kim D, Hu YJ, Zucker S, Cao J (2012). Transcriptional and epigenetic regulation of KIAA1199 gene expression in human breast cancer. PloS one.

[4] Matsuzaki S, Tanaka F, Mimori K, Tahara K, Inoue H, Mori M (2009). Clinicopathologic significance of KIAA1199 overexpression in human gastric cancer. Annals of surgical oncology.

[5] Sabates-Bellver J, Van der Flier LG, de Palo M, Cattaneo E, Maake C, Rehrauer H, Laczko E, Kurowski MA, Bujnicki JM, Menigatti M, Luz J, Ranalli TV, Gomes V, Pastorelli A, Faggiani R, Anti M (2007). Transcriptome profile of human colorectal adenomas. Molecular cancer research: MCR.

[6] Elvidge GP, Glenny L, Appelhoff RJ, Ratcliffe PJ, Ragoussis J, Gleadle JM (2006). Concordant regulation of gene expression by hypoxia and 2-oxoglutarate-dependent dioxygenase inhibition: the role of HIF-1alpha, HIF-2alpha, and other pathways. The Journal of biological chemistry.

[7] Brahimi-Horn MC, Chiche J, Pouyssegur J (2007). Hypoxia and cancer. J Mol Med (Berl).

[8] Harris AL (2002). Hypoxia—a key regulatory factor in tumour growth. Nature reviews Cancer.

[9] Ruan K, Song G, Ouyang G (2009). Role of hypoxia in the hallmarks of human cancer. J Cell Biochem.

[10] Jiang BH, Semenza GL, Bauer C, Marti HH (1996). Hypoxia-inducible factor 1 levels vary exponentially over a physiologically relevant range of O2 tension. Am J Physiol.

[11] Wang GL, Jiang BH, Rue EA, Semenza GL (1995). Hypoxia-inducible factor 1 is a basic-helix-loop-helix-PAS heterodimer regulated by cellular O2 tension. Proceedings of the National Academy of Sciences of the United States of America.

[12] Lee JW, Bae SH, Jeong JW, Kim SH, Kim KW (2004). Hypoxia-inducible factor (HIF-1)alpha: its protein stability and biological functions. Exp Mol Med.

[13] Dachs GU, Patterson AV, Firth JD, Ratcliffe PJ, Townsend KM, Stratford IJ, Harris AL (1997). Targeting gene expression to hypoxic tumor cells. Nat Med.

[14] Mole DR, Blancher C, Copley RR, Pollard PJ, Gleadle JM, Ragoussis J, Ratcliffe PJ (2009). Genome-wide association of hypoxia-inducible factor (HIF)-1alpha and HIF-2alpha DNA binding with expression profiling of hypoxia-inducible transcripts. The Journal of biological chemistry.

[15] Cohen I, Poreba E, Kamieniarz K, Schneider R (2011). Histone modifiers in cancer: friends or foes?. Genes Cancer.

[16] Johnson AB, Denko N, Barton MC (2008). Hypoxia induces a novel signature of chromatin modifications and global repression of transcription. Mutat Res.

[17] Watson JA, Watson CJ, McCann A, Baugh J (2010). Epigenetics, the epicenter of the hypoxic response. Epigenetics.

[18] Yang J, Ledaki I, Turley H, Gatter KC, Montero JC, Li JL, Harris AL (2009). Role of hypoxia-inducible factors in epigenetic regulation via histone demethylases. Ann N Y Acad Sci.

[19] Martin C, Zhang Y (2005). The diverse functions of histone lysine methylation. Nat Rev Mol Cell Biol.

[20] Zhou X, Sun H, Chen H, Zavadil J, Kluz T, Arita A, Costa M (2010). Hypoxia induces trimethylated H3 lysine 4 by inhibition of JARID1A demethylase. Cancer research.

[21] Klose RJ, Kallin EM, Zhang Y (2006). JmjC-domain-containing proteins and histone demethylation. Nat Rev Genet.

[22] Christensen J, Agger K, Cloos PA, Pasini D, Rose S, Sennels L, Rappsilber J, Hansen KH, Salcini AE, Helin K (2007). RBP2 belongs to a family of demethylases, specific for tri-and dimethylated lysine 4 on histone 3. Cell.

[23] Klose RJ, Yan Q, Tothova Z, Yamane K, Erdjument-Bromage H, Tempst P, Gilliland DG, Zhang Y, Kaelin WG (2007). The retinoblastoma binding protein RBP2 is an H3K4 demethylase. Cell.

[24] Birkenkamp-Demtroder K, Maghnouj A, Mansilla F, Thorsen K, Andersen CL, Oster B, Hahn S, Orntoft TF (2011). Repression of KIAA1199 attenuates Wnt-signalling and decreases the proliferation of colon cancer cells. British journal of cancer.

[25] Olive PL, Aquino-Parsons C, MacPhail SH, Liao SY, Raleigh JA, Lerman MI, Stanbridge EJ (2001). Carbonic anhydrase 9 as an endogenous marker for hypoxic cells in cervical cancer. Cancer research.

[26] Koukourakis MI, Bentzen SM, Giatromanolaki A, Wilson GD, Daley FM, Saunders MI, Dische S, Sivridis E, Harris AL (2006). Endogenous markers of two separate hypoxia response pathways (hypoxia inducible factor 2 alpha and carbonic anhydrase 9) are associated with radiotherapy failure in head and neck cancer patients recruited in the CHART randomized trial. Journal of clinical oncology : official journal of the American Society of Clinical Oncology.

[27] Sun X, Rogoff HA, Li CJ (2008). Asymmetric RNA duplexes mediate RNA interference in mammalian cells. Nature biotechnology.

[28] Sibley CR, Seow Y, Wood MJ (2010). Novel RNA-based strategies for therapeutic gene silencing. Molecular therapy : the journal of the American Society of Gene Therapy.

[29] van de Sluis B, Groot AJ, Vermeulen J, van der Wall E, van Diest PJ, Wijmenga C, Klomp LW, Vooijs M (2009). COMMD1 Promotes pVHL and O2-Independent Proteolysis of HIF-1alpha via HSP90/70. PloS one.

[30] Yan Q, Bartz S, Mao M, Li L, Kaelin WG (2007). The hypoxia-inducible factor 2alpha N-terminal and C-terminal transactivation domains cooperate to promote renal tumorigenesis *in vivo*. Mol Cell Biol.

[31] Liu S, Kumar SM, Martin JS, Yang R, Xu X (2011). Snail1 mediates hypoxia-induced melanoma progression. Am J Pathol.

[32] Maru S, Ishigaki Y, Shinohara N, Takata T, Tomosugi N, Nonomura K (2013). Inhibition of mTORC2 but not mTORC1 up-regulates E-cadherin expression and inhibits cell motility by blocking HIF-2alpha expression in human renal cell carcinoma. J Urol.

[33] Jami MS, Hou J, Liu M, Varney ML, Hassan H, Dong J, Geng L, Wang J, Yu F, Huang X, Peng H, Fu K, Li Y, Singh RK, Ding SJ (2014). Functional proteomic analysis reveals the involvement of KIAA1199 in breast cancer growth, motility and invasiveness. BMC cancer.

[34] Terashima M, Fujita Y, Togashi Y, Sakai K, De Velasco MA, Tomida S, Nishio K (2014). KIAA1199 interacts with glycogen phosphorylase kinase beta-subunit (PHKB) to promote glycogen breakdown and cancer cell survival. Oncotarget.

[35] Tiwari A, Schneider M, Fiorino A, Haider R, Okoniewski MJ, Roschitzki B, Uzozie A, Menigatti M, Jiricny J, Marra G (2013). Early insights into the function of KIAA1199, a markedly overexpressed protein in human colorectal tumors. PLoS One.

[36] Airley RE, Loncaster J, Raleigh JA, Harris AL, Davidson SE, Hunter RD, West CM, Stratford IJ (2003). GLUT-1 and CAIX as intrinsic markers of hypoxia in carcinoma of the cervix: relationship to pimonidazole binding. International journal of cancer Journal international du cancer.

[37] Chen J, Rocken C, Hoffmann J, Kruger S, Lendeckel U, Rocco A, Pastorekova S, Malfertheiner P, Ebert MP (2005). Expression of carbonic anhydrase 9 at the invasion front of gastric cancers. Gut.

[38] Sowter HM, Raval RR, Moore JW, Ratcliffe PJ, Harris AL (2003). Predominant role of hypoxia-inducible transcription factor (Hif)-1alpha versus Hif-2alpha in regulation of the transcriptional response to hypoxia. Cancer research.

[39] Tian H, McKnight SL, Russell DW (1997). Endothelial PAS domain protein 1 (EPAS1), a transcription factor selectively expressed in endothelial cells. Genes Dev.

[40] Iyer NV, Kotch LE, Agani F, Leung SW, Laughner E, Wenger RH, Gassmann M, Gearhart JD, Lawler AM, Yu AY, Semenza GL (1998). Cellular and developmental control of O2 homeostasis by hypoxia-inducible factor 1 alpha. Genes Dev.

[41] Tian H, Hammer RE, Matsumoto AM, Russell DW, McKnight SL (1998). The hypoxia-responsive transcription factor EPAS1 is essential for catecholamine homeostasis and protection against heart failure during embryonic development. Genes Dev.

[42] Koh MY, Lemos R, Liu X, Powis G (2011). The hypoxia-associated factor switches cells from HIF-1alpha- to HIF-2alpha-dependent signaling promoting stem cell characteristics, aggressive tumor growth and invasion. Cancer research.

[43] Xia X, Kung AL (2009). Preferential binding of HIF-1 to transcriptionally active loci determines cell-type specific response to hypoxia. Genome Biol.

[44] Chaturvedi CP, Somasundaram B, Singh K, Carpenedo RL, Stanford WL, Dilworth FJ, Brand M (2012). Maintenance of gene silencing by the coordinate action of the H3K9 methyltransferase G9a/KMT1C and the H3K4 demethylase Jarid1a/KDM5A. Proceedings of the National Academy of Sciences of the United States of America.

[45] Blagosklonny MV (2004). Antiangiogenic therapy and tumor progression. Cancer cell.

[46] Abe S, Usami S, Nakamura Y (2003). Mutations in the gene encoding KIAA1199 protein, an inner-ear protein expressed in Deiters' cells and the fibrocytes, as the cause of nonsyndromic hearing loss. J Hum Genet.

[47] Ui-Tei K, Naito Y, Takahashi F, Haraguchi T, Ohki-Hamazaki H, Juni A, Ueda R, Saigo K (2004). Guidelines for the selection of highly effective siRNA sequences for mammalian and chick RNA interference. Nucleic acids research.

[48] Pastorekova S, Parkkila S, Parkkila AK, Opavsky R, Zelnik V, Saarnio J, Pastorek J (1997). Carbonic anhydrase IX, MN/CA IX: analysis of stomach complementary DNA sequence and expression in human and rat alimentary tracts. Gastroenterology.

