# Supplementary material for: Hypoxia promotes colon cancer dissemination through up-regulation of cell migration-inducing protein (CEMIP)
Source: Oncotarget. 2015 May 13;6(24):20723–39. doi: 10.18632/oncotarget.3978 (PMC4653038; doi:10.18632/oncotarget.3978)
Supplement: Supplementary file 1 [file oncotarget-06-20723-s001.pdf]

SUPPLEMENTAL FIGURES AND TABLE

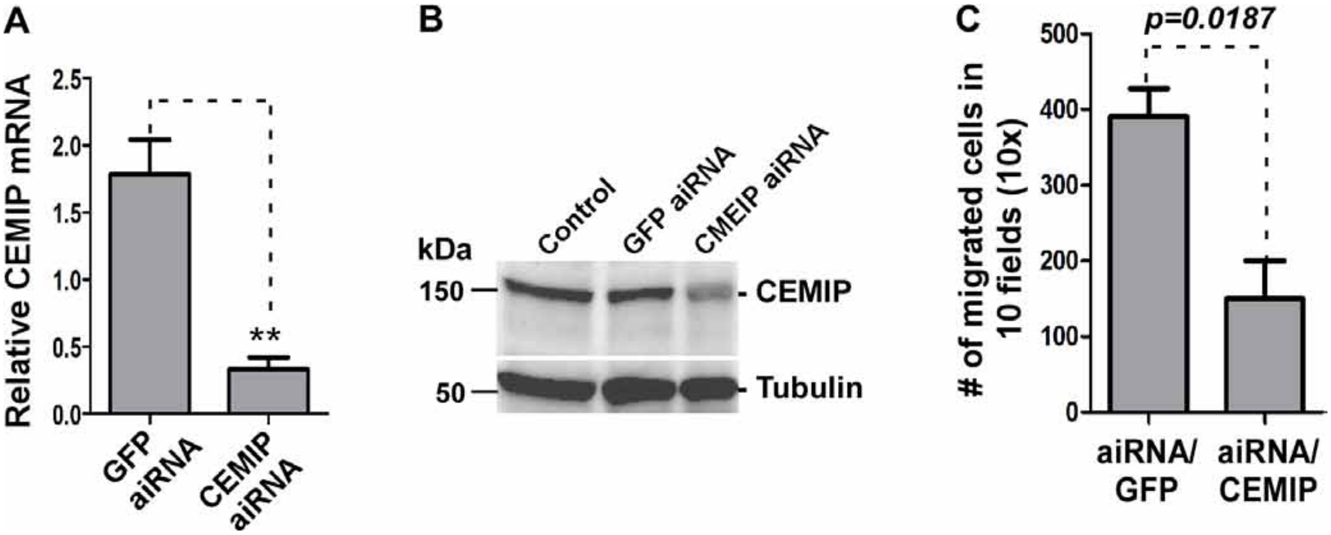

Supplementary Figure S1: Silencing of CEMIP in HCT-116 cells reduces HCT-116 cell migration. In the loss-of-function study, a commercially available aiRNA against CEMIP was employed. An asymmetric interfering RNA (aiRNA) is a novel approach used to silence gene expression. Total RNA isolated from human colon cancer HCT-116 cells transfected with aiRNAs against GFP control or CEMIP was analyzed by real time RT-PCR using CEMIP-specific primers. The expression was normalized using housekeeping gene HPRT-1. CEMIP aiRNA efficiently reduced endogenous CEMIP mRNA expression as compared to GFP aiRNA control **A.** Western blot analysis of whole cell lysates from HCT-116 control cells, GFP aiRNA or CEMIP aiRNA expressing cells using anti-CEMIP antibody. Tubulin was used as a loading control. aiRNA against CEMIP resulted in decreased CEMIP protein expression **B.** Transwell chamber migration assay was performed using HCT-116 cells transfected with aiRNA against GFP or CEMIP cells. Migrated cells were stained with Hoechst for nuclear staining and ten fields were examined using a Nikon 10x/0.25 NA lens. Silencing CEMIP in HCT-116 cells using aiRNA significantly reduced cell migration as compared to GFP aiRNA control **C.**

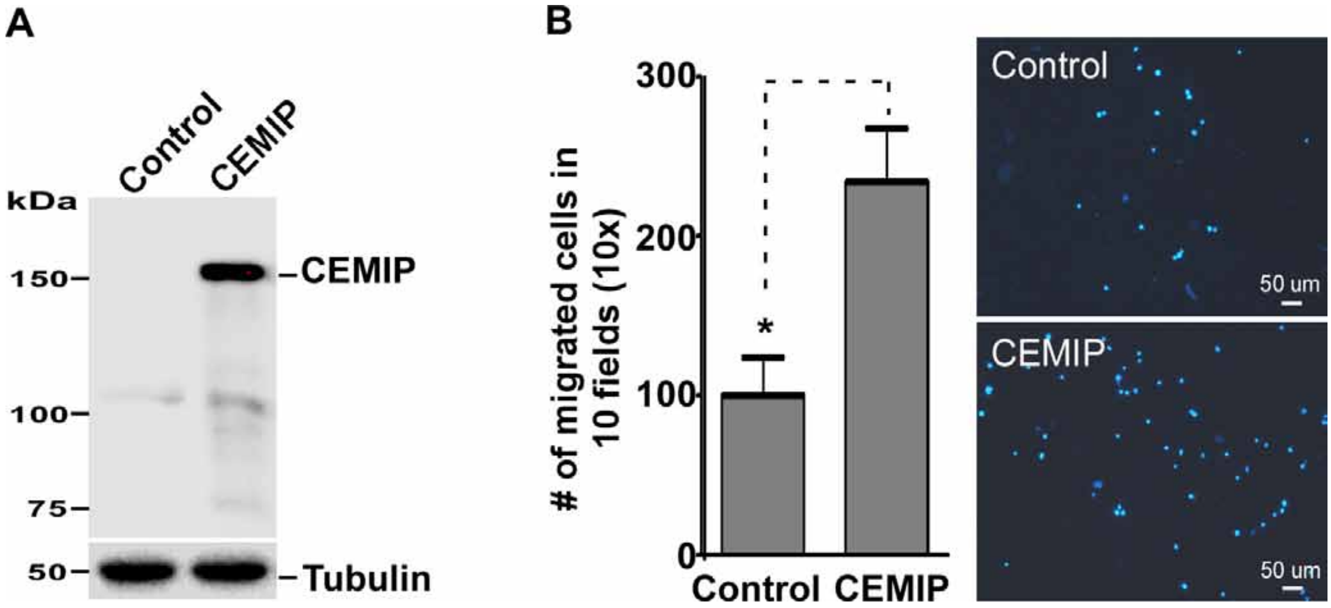

Supplementary Figure S2: Ectopic expression of CEMIP in SW480 cells results in enhanced cell migration. Minimally CEMIP-expressing SW480 cells were transiently transfected with CEMIP-Myc chimeric cDNA followed by Western blotting using anti-Myc antibody. CEMIP was detected only in the transfected SW480 cells **A.** Ectopic expression of CEMIP in SW480 cells resulted in increased migratory ability as examined by the Transwell chamber migration assay **B.**

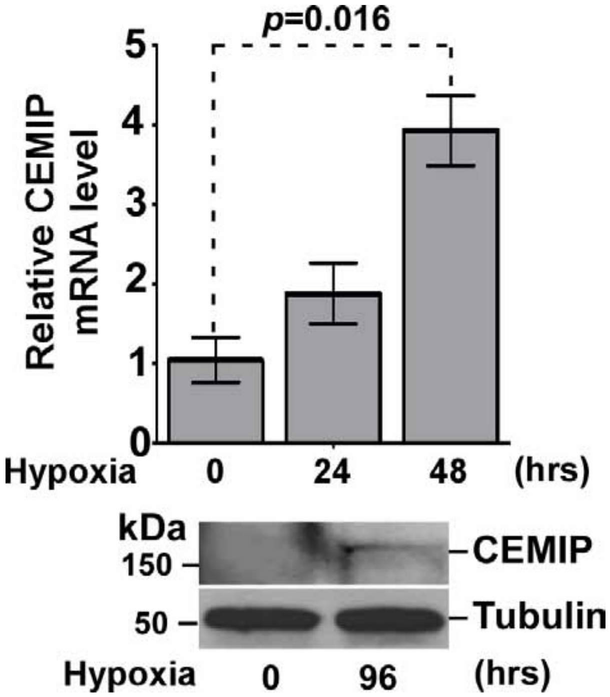

Supplementary Figure S3: Hypoxia induces CEMIP expression in HeLa cells. Real Time RT-PCR analysis of CEMIP mRNA expression in HeLa cells cultured under normoxic or hypoxic conditions for indicated times. The expression was normalized using housekeeping gene HPRT-1. Western blot analysis was performed in whole cells lysates from HeLa cells cultured under normoxic or hypoxic conditions for indicated times. Tubulin was used as a loading control.

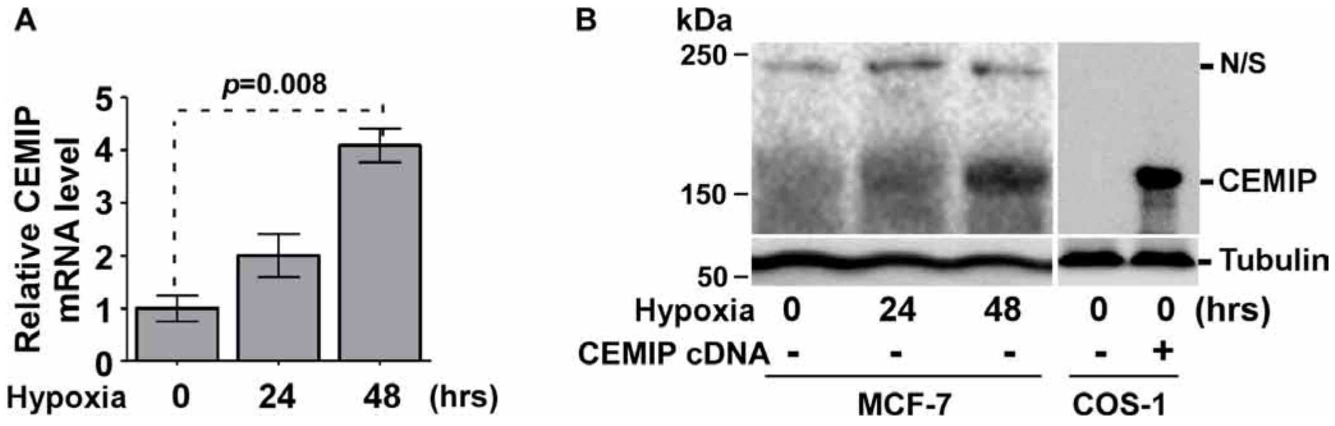

Supplementary Figure S4: Hypoxia induces CEMIP expression in MCF-7 cells. Total RNA isolated from MCF-7 cells cultured under hypoxic conditions for indicated time was examined by real time RT-PCR for CEMIP expression. Hypoxia gradually induced CEMIP mRNA expression **A.** The total cell lysates from MCF-7 cells cultured under hypoxic conditions for indicated time were examined by Western blotting using anti-CEMIP antibody. Western blotting of COS-1 cells transfected with CEMIP-Myc cDNA served as a control using anti-Myc antibody. Hypoxia induced CEMIP protein expression **B.**

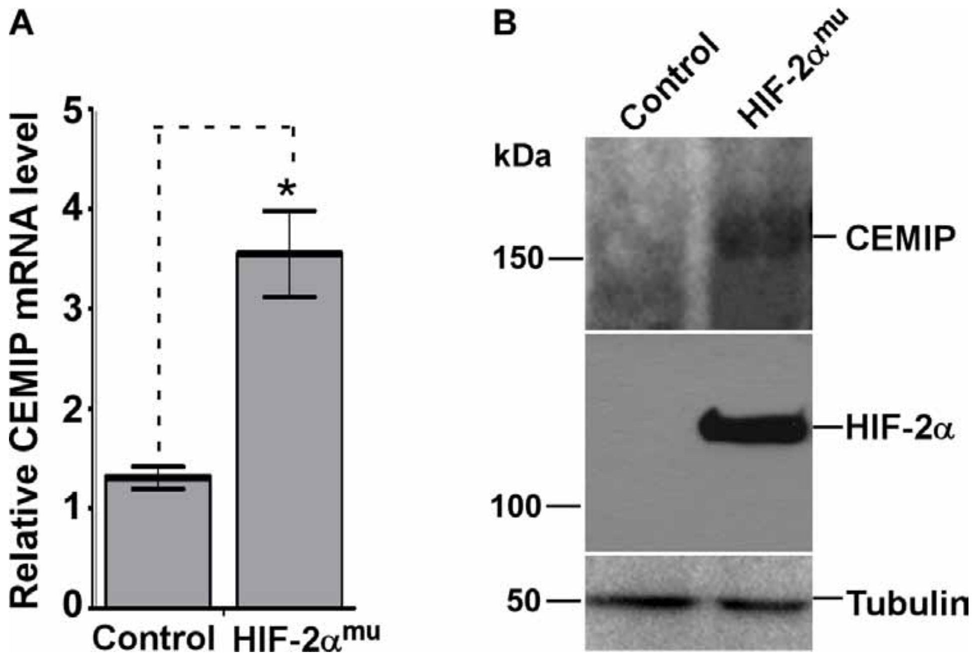

Supplementary Figure S5: HIF-2αincreases CEMIP expression in HeLa cells. Real Time RT-PCR analysis of CEMIP mRNA expression in HeLa cells transfected with vector (control) or HIF-2α^{mu} cDNA. The expression of CEMIP was normalized using housekeeping gene HPRT-1. HIF-2α^{mu} significantly induced CEMIP mRNA **A.** Total cell lysates of HeLa cells transfected with vector (control) or HIF-2α^{mu} cDNA were examined by Western blot using anti-CEMIP and anti-HIF-2αantibodies, respectively. Tubulin was used as a loading control **B.**

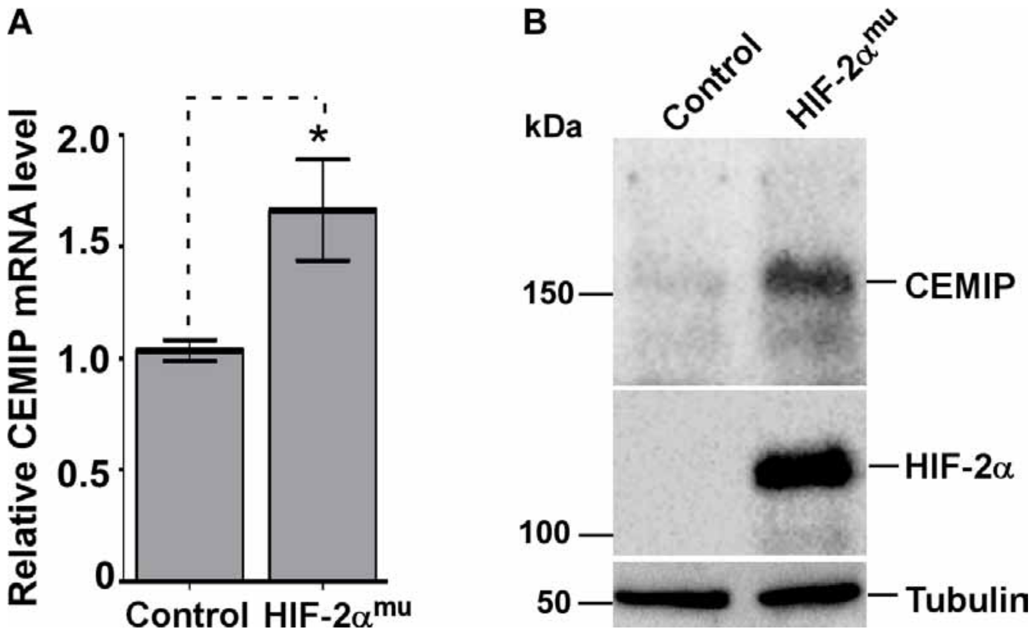

Supplementary Figure S6: HIF-2αincreases CEMIP expression in MCF-7 cells. Real Time RT-PCR analysis of CEMIP mRNA expression in MCF-7 cells transfected with vector (control) or HIF-2α^{mu} cDNA. The expression of CEMIP was normalized using housekeeping gene HPRT-1 **A.** Western blot analysis of whole cells lysates from MCF-7 cells transfected with vector (control) or HIF-2α^{mu} cDNA. Tubulin was used as a loading control **B.** HIF-2αinduced CEMIP expression at both the mRNA and protein level in MCF-7 cells.

Supplementary Table S1. Specification Sheet of Colon Cancer Tissue Array_CO1005
